# Reproductive and hormonal factors and bladder cancer risk: a prospective study and meta-analysis

**DOI:** 10.18632/aging.103523

**Published:** 2020-07-06

**Authors:** Xin Xu, Qiwang Mo, Haixiang Shen, Song Wang, Ben Liu

**Affiliations:** 1 Department of Urology, The First Affiliated Hospital, School of Medicine, Zhejiang University, Hangzhou 310003, Zhejiang, China

**Keywords:** bladder cancer, hormones, reproductive factors, PLCO, meta-analysis

## Abstract

Bladder cancer is three to four times more common among men than women. The objectives of this study were to explore the association between reproductive and hormonal factors and risk of bladder cancer among women using data from the Prostate, Lung, Colorectal and Ovarian (PLCO) cohort, and to perform a meta-analysis based on cohort studies. After a median of 11.6 years of follow-up, 237 incident bladder cancer cases were identified in PLCO cohort. Compared with menopause at 50-54 years, earlier menopause (< 45 years) was positively but not significantly associated with bladder cancer risk (HR 1.25, 95% CI 0.91-1.71; p = 0.176). In the meta-analysis, parous women had significantly lower bladder cancer risk than nulliparous women (pooled HR 0.79, 95% CI 0.73-0.86). In addition, menopause at an earlier age was significantly associated with a higher risk of bladder cancer (pooled HR 1.22, 95% CI 1.06-1.40). In conclusion, this study indicated a greater risk in bladder cancer among nulliparous women and among women with early menopause. Further studies are needed to understand the underlying mechanisms.

## INTRODUCTION

Incidence rates of bladder cancer differ notably between men and women. The estimated numbers of new bladder cancer cases in 2020 in the United States will be approximately 62,100 and 19,300 for men and women, respectively [1]. Smoking and occupational exposure to aromatic amines are the most established risk factors for bladder cancer [2], which are unlikely to fully explain discrepancies in incidence of bladder cancer between men and women.

Animal and human-cell preclinical studies have indicated that steroid hormone receptor signaling plays an important role in bladder cancer development and progression [3–5]. Emerging observational studies have investigated the potential relationship between hormonal and reproductive factors and bladder cancer risk in women with inconsistent results [6–9]. Understanding the effects of hormonal and reproductive factors on bladder cancer risk may contribute to its early detection, potentially improving prognosis. The objectives of this study were, therefore, to assess the association between hormones and reproductive factors and the risk of bladder cancer using data from the Prostate, Lung, Colorectal and Ovarian (PLCO) cohort, and to perform a meta-analysis based on all available prospective studies.

## RESULTS

After a median of 11.6 years of follow-up, 237 incident bladder cancer cases were identified in PLCO cohort. Table 1 shows the baseline participant characteristics according to occurrence of bladder cancer. Generally, bladder cancer cases were older, less educated, more often of white race, and much more likely to be ever smokers, compared to non-cases. There was little difference in other characteristics, including BMI, marital status, alcohol drinking habits and family history of cancer, between the two groups.

**Table 1:** Baseline participant characteristics according to bladder cancer occurrence in the PLCO cohort.

**Variables**	**Non-cases (n=70,347)**	**Cases (n=237)**	**p-value**
Control group (n, %)	35,129 (49.9%)	115 (48.5%)	0.664
Age (years), mean (SD)	62.47 ± 5.38	63.77 ± 5.15	<0.001
Smoking status (n, %)			<0.001
Never	39,485 (56.1%)	79 (33.3%)	
Current	6,718 (9.5%)	48 (20.3%)	
Former	24,135 (34.3%)	110 (46.4%)	
Missing	9 (0.0%)	0 (0.0%)	
Education (n, %)			0.020
≤High school	33,063 (47.0%)	127 (53.6%)	
≥Some college	37,108 (52.7%)	108 (45.6%)	
Missing	176 (0.3%)	2 (0.8%)	
Body mass index (n, %)			0.617
<25.0 kg/m2	27,984 (39.8%)	101 (42.6%)	
≥25.0 kg/m2	41,354 (58.8%)	132 (55.7%)	
Missing	1,009 (1.4%)	4 (1.7%)	
Race (n, %)			0.014
White, Non-Hispanic	62,207 (88.4%)	224 (94.5%)	
Other	8,114 (11.5%)	13 (5.5%)	
Missing	26 (0.0%)	0 (0.0%)	
Marital status (n, %)			0.083
Married	48,590 (69.1%)	156 (65.8%)	
Not married	21,601 (30.7%)	79 (33.3%)	
Missing	156 (0.2%)	2 (0.8%)	
Family history of cancer (n, %)	41,515 (59.2%)	145 (61.4%)	0.490
Alcohol drinking status (n, %)			0.277
Never	7,493 (10.7%)	21 (8.9%)	
Former	7,937 (11.3%)	22 (9.3%)	
Current	38,340 (54.5%)	144 (60.8%)	
Missing	16,577 (23.6%)	50 (21.1%)	

PLCO, prostate, lung, colorectal and ovarian; SD, standard deviation

Table 2 shows the HRs for bladder cancer according to hormonal and reproductive factors in the PLCO cohort. Early menopause (< 45 years) was positively but not significantly associated with bladder cancer risk, compared with menopause at 50-54 years (adjusted HR 1.25, 95% CI 0.91-1.71; p = 0.176). A nonsignificant elevation in risk for bladder cancer was found in women with HRT use compared with non-users (adjusted HR 1.32, 95% CI 0.99-1.76; p = 0.057), with the greatest risk in women who reported HRT use ≥ 10 years (adjusted HR 1.50, 95% CI 1.08-2.09; p = 0.017). Neither the use of oral contraceptive (OC) nor the duration of OC use was related to bladder cancer risk. No significant associations were observed for history of hysterectomy or oophorectomy. However, a borderline statistically significant inverse association was observed for women who underwent hysterectomy at age ≥ 50 years compared with women who had hysterectomy at age < 40 years (adjusted HR 0.46, 95% CI 0.21-1.00; p = 0.051). There was no significant association between bladder cancer and age at menarche, parity, age at first birth, number of live births, stillbirth, and miscarriages, either in crude or multivariable-adjusted analyses. All of above associations were not modified by smoking status (p for interaction > 0.05).

**Table 2:** Association between hormonal and reproductive factors and bladder cancer risk in the PLCO cohort.

**Factors**	**Cohort (n)**	**Cases (n)**	**Crude HR (95% CI), *p***	Multi-adjusted HR (95% CI)*, *p*
Age at menarche, y				
≤11	14,263	50	Reference	Reference
12–13	37,820	119	0.88 (0.64-1.23), p=0.467	0.86 (0.62-1.20), p=0.386
14–15	15,101	54	1.00 (0.68-1.47), p=0.998	0.95 (0.64-1.40), p=0.787
≥16	3,206	13	1.15 (0.62-2.11), p=0.656	1.09 (0.59-2.00), p=0.794
Parity				
Nulliparous	6,452	20	Reference	Reference
Parous	64,021	217	1.07 (0.68-1.69), p=0.768	1.03 (0.65-1.63), p=0.903
No. of live births				
0	6,452	20	Reference	Reference
1–2	21,892	72	1.05 (0.64-1.73), p=0.833	1.05 (0.64-1.72), p=0.849
3–4	29,254	102	1.09 (0.68-1.77), p=0.714	1.04 (0.64-1.68), p=0.873
≥5	12,875	43	1.05 (0.62-1.78), p=0.863	0.97 (0.57-1.65), p=0.904
Age at first live birth, y				
<20	12,059	36	0.81 (0.56-1.17), p=0.256	0.77 (0.53-1.13), p=0.177
20–24	32,763	126	Reference	Reference
25–29	14,113	40	0.73 (0.51-1.04), p=0.083	0.76 (0.53-1.08), p=0.127
≥30	4,868	13	0.69 (0.39-1.22), p=0.205	0.73 (0.41-1.30), p=0.284
Ever had stillbirth				
0	66,986	230	Reference	Reference
1	2,478	5	0.60 (0.25-1.45), p=0.256	0.56 (0.23-1.36), p=0.203
≥2	598	2	1.03 (0.26-4.15), p=0.965	1.02 (0.25-4.09), p=0.983
No. of miscarriages				
0	46,249	153	Reference	Reference
1	15,520	56	1.09 (0.80-1.48), p=0.586	1.07 (0.78-1.45), p=0.682
≥2	8,575	28	0.99 (0.66-1.48), p=0.965	0.94 (0.63-1.40), p=0.751
Age at menopause, y				
<45	19,607	76	1.32 (0.96-1.81), p=0.088	1.25 (0.91-1.71), p=0.176
45–49	16,675	57	1.14 (0.81-1.60), p=0.457	1.08 (0.77-1.52), p=0.658
50–54	25,748	78	Reference	Reference
≥55	7,942	25	1.04 (0.66-1.62), p=0.88	1.14 (0.73-1.80), p=0.557
Type of Menopause				
Natural Menopause	43,571	151	Reference	Reference
Surgery	23,314	80	1.02 (0.77-1.33), p=0.907	1.06 (0.81-1.39), p=0.666
Drug Therapy	2,325	4	0.50 (0.19-1.35). p=0.171	0.59 (0.22-1.60). p=0.301
History of hysterectomy				
No	45,410	153	Reference	Reference
Yes	25,000	84	1.02 (0.78-1.34), p=0.866	1.05 (0.80-1.37), p=0.738
Age at hysterectomy, y				
<40	8,780	31	Reference	Reference
40–44	6,121	23	1.06 (0.62-1.81), p=0.837	1.02 (0.59-1.76), p=0.939
45–49	5,164	22	1.19 (0.69-2.05), p=0.537	1.20 (0.69-2.08), p=0.515
≥50	4,843	8	0.46 (0.21-1.01), p=0.052	0.46 (0.21-1.00), p=0.051
Oophorectomy status				
No	55,379	190	Reference	Reference
Yes	13,936	42	0.92 (0.66-1.28), p=0.608	0.92 (0.66-1.29), p=0.631
OC use				
Never	32,178	112	Reference	Reference
Ever	38,279	125	0.95 (0.74-1.23), p=0.711	1.07 (0.81-1.40), p=0.639
Duration of OC use, y				
Never	32,166	112	Reference	Reference
1–3	17,862	62	1.01 (0.74-1.38), p=0.939	1.13 (0.82-1.56), p=0.471
4–5	5,205	13	0.73 (0.41-1.30), p=0.286	0.87 (0.48-1.55), p=0.628
6–9	6,300	22	1.02 (0.64-1.61), p=0.940	1.15 (0.72-1.84), p=0.554
≥10	8,835	27	0.89 (0.58-1.35), p=0.586	0.96 (0.62-1.48), p=0.850
HRT use				
Never	22,921	69	Reference	Reference
Ever	47,062	168	1.18 (0.89-1.56), p=0.243	1.32 (0.99-1.76), p=0.057
Duration of HRT use, y				
Never	22,921	69	Reference	Reference
≤5	21,299	74	1.14 (0.82-1.59), p=0.425	1.30 (0.93-1.81), p=0.125
6–9	8,668	20	0.76 (0.46-1.24), p=0.271	0.92 (0.55-1.53), p=0.740
≥10	17,095	74	1.45 (1.05-2.02), p=0.026	1.50 (1.08-2.09), p=0.017

*Adjusted for age (categorical), race (non-Hispanic white vs. Other), body mass index (<25 kg/m^2^ vs. ≥25 kg/m^2^), education (≤high school vs. ≥some college), and smoking status (never vs. former ≤ 15 years since quit vs. former > 15 years since quit vs. former year since quit unknown vs. current smoker ≤ 1 pack per day vs. current smoker >1 pack per day vs. current smoker intensity unknown).

PLCO, prostate, lung, colorectal and ovarian; HR, hazard ratio; CI, confidence interval; y, year; No., number; OC, oral contraceptive; HRT, hormone replacement therapy.

**Figure 1:**
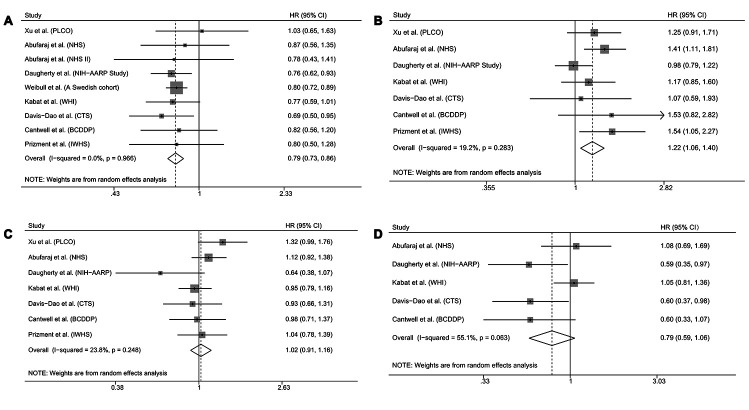
**Forest plots showing risk estimates of the association between bladder cancer and reproductive and hormonal factors.** Risk estimates for bladder cancer and parity (**A**); early menopause (**B**); any use of hormone replacement therapy (**C**); use of estrogen-progestogen therapy for hormone replacement therapy (**D**). The square denotes the weight size of each included study and the diamond represents the pooled HR (95% CI).

Figure 1 shows risk estimates of the association between bladder cancer and reproductive and hormonal factors in the meta-analysis. We can see that parous women had significantly lower bladder cancer risk than nulliparous women (pooled HR 0.79, 95% CI 0.73-0.86; Figure 1A). No significant heterogeneity was observed between the study-specific HRs overall (p for heterogeneity = 0.966). In addition, we observed a significant association between menopause at an earlier age and bladder cancer risk (pooled HR 1.22, 95% CI 1.06-1.40; Figure 1B) without obvious heterogeneity between studies (p for heterogeneity = 0.283). No significant association was found for any use of hormone replacement therapy (HRT) (pooled HR 1.02, 95% CI 0.91-1.16; Figure 1C). However, women who used estrogen-progestogen therapy (EPT) for HRT experienced a no significantly lower risk than those who never used HRT (pooled HR 0.79, 95% CI 0.59-1.06; Figure 1D).

## DISCUSSION

In this meta-analysis based on the PLCO cohort and seven previously published prospective studies, we observed a higher risk of bladder cancer among nulliparous women compared with parous women and among women who experienced early menopause. No association was observed for any use of HRT, although a no significantly lower risk was found among women using EPT.

Although the findings of PLCO cohort did not support an inverse association between parity and bladder cancer, parity has been shown to be negatively associated with bladder cancer risk in various observational studies [7–11]. Most of these studies did not find a downward trend in risk with increasing number of births, which indicated that there may be a threshold effect in which the protective mechanism underlying parity was established after the first birth. Pregnant women experience dramatic increases in estrogen and progesterone levels; however, the exact mechanisms through which estrogen and progesterone affect lifetime bladder cancer risk remain unclear. Hoffman et al. [12] found that raloxifene inhibited growth of bladder cancer cells via estrogen receptor-dependent induction of apoptosis and inhibition of proliferation. Estrogen receptor alpha (ERα) was shown to play a protective role in bladder cancer through circ_0023642/miR-490-5p/EGFR signaling [13]. Studies using bladder cancer tissue specimens have demonstrated that elevated or reduced expression of estrogen and progesterone receptors as well as alterations of their upstream or downstream pathways are associated with clinical outcomes [14, 15].

Early age at menopause has been associated with a higher risk of bladder cancer in several previous cohort studies [6, 11, 16] and the present PLCO cohort, although not all associations were statistically significant. In addition, we observed a over 50% decrease in risk for bladder cancer among women who reported age at hysterectomy ≥50 years compared with women who reported age at hysterectomy <40 years in the PLCO cohort, which indirectly supported that early age at menopause was associated with an increased risk of bladder cancer. Of note, this finding could be confounded by smoking status as a previous study showed that smoking women experience an earlier age at menopause than non-smokers [17]. However, in this analysis the result did not differ after adjusting for smoking status.

Many prospective studies, including the PLCO cohort, have either found a null association or a positive association between bladder cancer and HRT [6, 11, 16]. Findings from our meta-analysis based on seven cohort studies suggested that HRT was not related to the risk of bladder cancer. However, a few previous cohort studies have indicated that bladder cancer risk may differ by formulation and reported a potential inverse association with EPT use [8, 9]. Therefore, we also performed a meta-analysis and found that EPT was negatively but not significantly associated with bladder cancer. McGrath et al. [18] reported in 2006 that women who used EPT for HRT experienced a no significantly lower risk than those who never used HRT in the Nurses’ Health Study (NHS) cohort. However, an updated NHS study in 2020 failed to find an inverse association between EPT and bladder cancer risk with ten years of additional follow-up and double the number of cases [6]. Therefore, the association between EPT and bladder cancer is still unclear and should be further examined in future prospective studies.

Strengths of the PLCO study included the large size, the population-based setting, a comprehensive list of potential confounders, and a virtually complete follow-up. Strengths of our meta-analysis included the strict inclusion criteria (restriction to prospective studies), the large sample size, and the homogeneity across eligible studies. Several limitations should also be mentioned. First, only baseline exposure data were used in the PLCO analysis and thus we were not able to take into account change in exposure status over time. Second, the results were still potentially biased by residual confounding after multi-adjustment. In the PLCO cohort, we could not adjust our results for exposures to chemicals and physical activity. Third, a meta-analysis is unable to solve problems with confounding variables that could be inherent in the original studies and inadequate control of all known confounders may bias the summary results. Finally, the PLCO and meta-analysis have some contradicting results. For example, menopause at an earlier age was significantly associated with a higher risk of bladder cancer in the meta-analysis. On the other hand, an earlier menopause was positively but not significantly associated with bladder cancer risk in the PLCO cohort, which may have limited sample size and statistical power compared with a meta-analysis.

In conclusion, our study indicated an elevation in risk for bladder cancer among nulliparous women and among women who reported earlier age at menopause. The underlying mechanisms that may be responsible for these relationships remain to be determined.

## MATERIALS AND METHODS

### Subjects and study design

The design and methods of the PLCO screening trial have been published [19]. Briefly, the PLCO study is a large-scale clinical trial designed to determine whether certain screening tests reduce death from prostate, lung, colorectal, and ovarian cancer. A total of 154,897 eligible participants between 55 and 74 years were enrolled into the PLCO trial at 10 clinical screening centers throughout the United States between November 1993 and July 2001. Reproductive and hormonal factors, including age at menarche, number of live-born children, age at first birth, age at menopause, reason for menopause, hysterectomy status, oophorectomy status, OC use, and HRT, were ascertained on the baseline questionnaire. Age, sex, race, education, marital status, smoking status, family history of cancer, height, and weight were also collected in the baseline questionnaire. PLCO study was approved by the institutional review boards of the National Cancer Institute and each of the participating centers. Informed consent was obtained from each eligible participant in the study.

### Subject selection and bladder cancer case ascertainment

In this study, participants were excluded if they were male (n = 76,682); had not returned a baseline questionnaire (n = 2,094); had reported a previous cancer at baseline (n = 5,168); had died of an unknown cause or had an undetermined case status (n = 36); or did not have follow-up time (n = 333). Thus, the cohort for analysis consisted of 70,584 women.

Incident cases of primary carcinoma of the urinary bladder were updated annually using a self-reported questionnaire. Participants were asked whether they had been diagnosed with any cancer, cancer types, and diagnosis date in the previous year. State registries, death certificates and physician reports were examined as additional sources of cancer incidence data. Cancer diagnoses were verified through medical record abstraction. Participants were followed until cancer diagnosis or death, or end of follow-up (December 31, 2009).

### Statistical analysis

A multivariate Cox proportional hazards model was used to estimate hazard ratios (HRs) and 95% confidence intervals (CIs). Models were adjusted for potential confounders including age (categorical), race (non-Hispanic White vs. Other), body mass index (BMI, < 25 kg/m^2^ vs. ≥ 25 kg/m^2^), education (≤ high school vs. ≥ some college), and smoking status (never vs. former ≤ 15 years since quit vs. former > 15 years since quit vs. former year since quit unknown vs. current smoker ≤ 1 pack per day vs. current smoker > 1 pack per day vs. current smoker intensity unknown). Tests of multiplicative interaction were performed using likelihood-ratio tests compared models with and without the interaction term. The proportional hazards (PH) assumption was examined using the Schoenfeld residual test [20].

### Meta-analysis

Literature search: A comprehensive literature search and selection was performed in PubMed through February 2020 by two independent reviewers (X.X. and B.L.).

### Inclusion criteria

(*i*) investigated the associations between hormonal and/or reproductive exposures and bladder cancer risk, (*ii*) risk estimates with their 95 % CIs were given or sufficient information was provided for calculation, and (*iii*) the study design was cohort, nested case-control, case-cohort or clinical trial. Seven previously published prospective studies [6–11, 16] and the PLCO cohort were finally included in the meta-analysis.

### Statistical methods

We analyzed the effects of ever versus never exposure to parity, age at menopause, use of any HRT, and use of EPT for HRT on bladder cancer risk using a meta-analysis approach, because these associations reported in published literature were inconsistent. If a single study reported results for different exposures (e.g., different number of births) but did not report the overall results, we combined the corresponding estimates using the methods proposed by Hamling et al. [21], taking into account the correlation between estimates. A random effects model [22] was used to calculate summary HRs and 95 % CIs. Heterogeneity among studies was assessed by the Q test and I^2^. All statistical analyses were performed using the software STATA version 15 (Stata Corp, College Station, TX, USA). A two-sided P-value <0.05 was considered significant.
